# Draft Genome Sequences of Clostridium tyrobutyricum Strains FAM22552 and FAM22553, Isolated from Swiss Semihard Red-Smear Cheese

**DOI:** 10.1128/genomeA.00078-15

**Published:** 2015-03-12

**Authors:** Michelangelo Storari, Daniel Wüthrich, Rémy Bruggmann, Hélène Berthoud, Emmanuelle Arias-Roth

**Affiliations:** a Institute for Food Sciences, Agroscope, Bern, Switzerland; b Interfaculty Bioinformatics Unit, University of Bern and Swiss Institute of Bioinformatics, Bern, Switzerland

## Abstract

Clostridium tyrobutyricum is the main microorganism responsible for late blowing defect in cheeses. Here, we present the draft genome sequences of two C. tyrobutyricum strains isolated from a Swiss semihard red-smear cheese. The two draft genomes comprise 3.05 and 3.08 Mbp and contain 3,030 and 3,089 putative coding sequences, respectively.

## GENOME ANNOUNCEMENT

The spore-forming anaerobic bacterium Clostridium tyrobutyricum is the main cause of late blowing defect in hard and semihard cheeses (1). Clostridium spores contaminate milk during milking and survive the pasteurization process. The growth of C. tyrobutyricum during cheese maturation causes the formation of butyric acid, carbon dioxide, and molecular hydrogen, leading to the loss of the commercial value of the final product (2). The primary metabolism of C. tyrobutyricum as well as its nutritional requirements are still almost unknown, despite the huge negative economic impact that this microorganism has on the cheese manufacturing industry. To date, only the genomes of a strain used in industrial butyric acid production and a strain isolated from Grana Padano cheese have been sequenced (3, 4).

In the present study, we report the draft genome sequences of two C. tyrobutyricum strains (FAM22552 and FAM22553; deposited in the culture collection of Agroscope, Bern, Switzerland) isolated from a Swiss semihard red-smear cheese with typical symptoms of late blowing in 2012. Genomic DNA was extracted from liquid cultures of the two C. tyrobutyricum strains using the EZ1 DNA tissue kit (Qiagen) and sent for sequencing to Microsynth AG (Switzerland). Libraries were prepared with the Nextera XT DNA sample preparation kit (Illumina) and sequenced using the Illumina MiSeq sequencing platform. Low-quality bases of raw reads (2 × 301 bp) were removed using Trimmomatic (version 0.30, SLIDINGWINDOW:4:15 MINLEN:127 [5]) before *de novo* assembly was performed. *De novo* assembly was carried out using SPAdes version 3.1.0 (*k*-mer sizes of 21, 33, 55, 77, 99, and 127 [6]). The obtained contigs were assembled to scaffolds using SSPACE version 3.0 (default parameters [7]). Trimmed reads (Trimmomatic, SLIDINGWINDOW:4:15 MINLEN:101 CROP:101) were mapped to the scaffolds with Bowtie2 version 2.1.0 (default parameters [8]) to determine the depth of coverage using SAMtools version 0.1.19 (idxstats [9]). Scaffolds with a mean depth of coverage lower than 20% of the mean depth of coverage of all scaffolds as well as contigs shorter than 200 bp were discarded. The *de novo* assembly consists of 58 (3.05 Mbp) and 62 (3.08 Mbp) scaffolds for FAM22552 and FAM22553, respectively. Assembled genomes were annotated using the RAST server (10). In total, 3,030 putative coding sequences were annotated for FAM22552 and 3,089 for FAM22553.

C. tyrobutyricum grows in cheese using lactate and acetate as carbon and energy sources (11). A preliminary analysis of the two annotated genomes revealed the presence of putative genes coding for many enzymes needed to convert these two compounds into butyric acid, carbon dioxide, and molecular hydrogen. These include l- and d-lactate dehydrogenases, pyruvate-ferredoxin oxidoreductases, thiolases, hydroxybutyryl-CoA dehydrogenases, crotonases, hydroxybutyryl-CoA dehydrogenases, phosphotransacetylases, and acetate kinases (for a review, see reference 12). Studies are now under way to further clarify the metabolism of C. tyrobutyricum in cheese.

### Nucleotide sequence accession numbers.

The whole-genome shotgun projects have been deposited at DDBJ/EMBL/GenBank under the accession numbers JTER00000000 (for C. tyrobutyricum FAM22552) and JTES00000000 (for C. tyrobutyricum FAM22553). The versions described in this paper are the versions JTER01000000 and JTES01000000.
